# Biomaterials-Enhanced Intranasal Delivery of Drugs as a Direct Route for Brain Targeting

**DOI:** 10.3390/ijms24043390

**Published:** 2023-02-08

**Authors:** Elena Marcello, Valeria Chiono

**Affiliations:** 1 Department of Mechanical and Aerospace Engineering, Politecnico di Torino, Corso Duca Degli Abruzzi 24, 10129 Turin, Italy; 2 Interuniversity Center for the Promotion of 3Rs Principles in Teaching and Research, Centro 3R, 56122 Pisa, Italy; 3 Institute for Chemical-Physical Processes, National Research Council (CNR-IPCF), 56124 Pisa, Italy

**Keywords:** brain targeting, drug delivery, intranasal, hydrogels, nanoparticles

## Abstract

Intranasal (IN) drug delivery is a non-invasive and effective route for the administration of drugs to the brain at pharmacologically relevant concentrations, bypassing the blood–brain barrier (BBB) and minimizing adverse side effects. IN drug delivery can be particularly promising for the treatment of neurodegenerative diseases. The drug delivery mechanism involves the initial drug penetration through the nasal epithelial barrier, followed by drug diffusion in the perivascular or perineural spaces along the olfactory or trigeminal nerves, and final extracellular diffusion throughout the brain. A part of the drug may be lost by drainage through the lymphatic system, while a part may even enter the systemic circulation and reach the brain by crossing the BBB. Alternatively, drugs can be directly transported to the brain by axons of the olfactory nerve. To improve the effectiveness of drug delivery to the brain by the IN route, various types of nanocarriers and hydrogels and their combinations have been proposed. This review paper analyzes the main biomaterials-based strategies to enhance IN drug delivery to the brain, outlining unsolved challenges and proposing ways to address them.

## 1. Introduction

Pathologies affecting the brain tissue, such as neurodegenerative diseases, brain tumors and stroke, need the local administration of drugs for treatment. However, the effectiveness of drug administration to the brain is hampered by the presence of the blood–brain barrier (BBB), which is the biological barrier that regulates the transport of molecules between the blood and the brain. The BBB is constituted by the brain microvascular endothelium (formed by a monolayer of endothelial cells) with its basement membrane, pericytes surrounding the endothelium and astrocytes mediating the interaction between neuronal cells and oligodendrocytes with the brain capillaries. The BBB has a selective permeability, which is fundamental for normal brain physiology [1,2]. The interplay among endothelial cells, pericytes and astrocytes regulates BBB integrity in vivo as well as in vitro [3,4]. Hence, the BBB protects the brain from the entry of potentially toxic substances, however, it also prevents the delivery of therapeutics into the central nervous system (CNS) for disease treatment. The BBB shows a low level of pinocytosis and possesses tight junctions, which form a seal between opposing endothelial membranes. The presence of tight junctions causes a high transendothelial electrical resistance of 1500–2000 Ω·cm^2^ compared to 3–30 Ω·cm^2^ in the peripheral microvasculature [5]. For these reasons, the BBB highly restricts paracellular diffusion of solutes from the blood into the brain. Typically, only small lipophilic molecules may cross the BBB via transcellular passive diffusion, although some limited transport of certain peptides and peptide analogs has been reported [6]. Transcellular active diffusion occurs through specific transporters or receptors, such as glucose transporter 1 for glucose and transferrin receptor for iron [2,5]. In addition, receptors and transporters for gastrointestinal hormones involved in regulating metabolism are expressed at the BBB in order to convey information between CNS and peripheral parts of the body [5]. Besides the low paracellular diffusion and low rate of pinocytosis, the endothelial layer of the BBB is also provided with efflux pumps (such as P-glycoprotein) which further restrict the entry of substances that would be otherwise predicted to cross the BBB based on their chemical characteristics and molecular weight [7].

**Figure 1:**
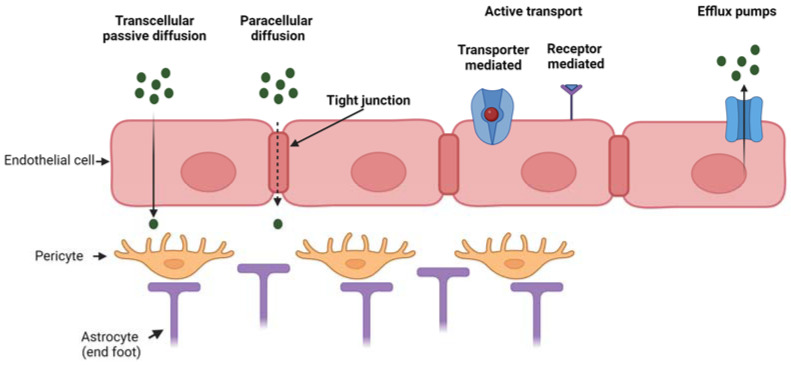
Mechanism of transport through the BBB which is composed of three main cell types (endothelial cells, pericytes and astrocytes). Passive transport through transcellular or paracellular diffusion, active transport (either transporter- or receptor-mediated) and efflux active pumps. Created with BioRender.com.

Figure 1 reports the main transport mechanisms across the endothelial layer of the BBB.

Although a few low molecular weight drugs can cross the BBB, high molecular weight hydrophilic substances are severely restricted from crossing the BBB under normal conditions. Intraparenchymal, intracerebroventricular and intrathecal injections/infusions can be used to directly deliver therapeutics into the CNS [8], but these routes of administration are invasive and likely not practical for drugs which need to be given frequently for the treatment of chronic diseases. 

Additional invasive methods include the temporary disruption of BBB integrity, e.g., by osmotic shock to the endothelial layer by mannitol administration or by an ultrasound disruption technique [8]. Such methods are costly, require long-term hospitalization and are associated with several drawbacks related to enhanced BBB permeability, such as neuron damage by cytotoxic molecules crossing the BBB. 

Alternative strategies include a chemical modification of drugs to enhance their lipophilicity without affecting their activity or the introduction of hydrophilic molecules into nanomicelles with a hydrophobic shell, e.g., based on polaxamer-type copolymers [8]. Further “physiological approaches” are possible exploiting the mechanisms involved in the transport of metabolites and catabolites across the BBB, i.e., transcytosis mechanisms [8]. For example, it is possible to incorporate drugs within nanocarriers surface functionalized with ligands interacting with insulin or transferrin receptors on the endothelial cell layer of the BBB. One main disadvantage is that such transcytosis mechanisms are not specific of the BBB, therefore part of the drug-loaded nanocarriers may reach different tissues than the CNS with possible side effects. Moreover, systemic delivery of drug-loaded nanocarriers to reach the brain by intravenous or oral administration is challenging due to the hepatic first pass metabolism effect reducing drug half-life [9]. 

Hence, intranasal (IN) delivery has emerged as a non-invasive and direct route for drug administration to the brain bypassing the BBB and allowing rapid brain localization [10]. The benefits afforded by IN delivery, especially the removal of systemic side effects and the possibility to deliver biologics to the brain (peptides, proteins, oligonucleotides and even cells), places it as a potentially powerful route for brain disorder treatment [10]. However, the main limitations of IN delivery of drugs include: (i) loss of non-absorbed drugs in the respiratory and digestive tracts with potential side effects; (ii) rapid muco-ciliary clearance increasing drug loss; (iii) low nasal epithelium permeability for macromolecular and hydrophilic drugs; (iv) nasal mucosa metabolism of drugs by proteolytic enzymes. To bypass these limitations, new advanced IN drug delivery systems are required with the following characteristics: (i) high encapsulation efficiency, (ii) ability to protect the drugs from degradation/denaturation, (iii) ability to favor drug retention at the nasal mucosa and (iv) ability to promote drug transport through the nasal epithelium to reach the brain tissue. Several types of nanoparticles and hydrogels have been developed to achieve these aims in order to increase the effectiveness of the drug transport by the IN route. This review is intended to explain the general mechanism for drug delivery to the brain by the IN route, and how this has been enhanced by the design and use of nanoparticles and hydrogels as drug carriers. Research efforts aimed at improving efficiency of IN drug carrier systems are discussed.

## 2. Overview of Nasal Anatomy

Since ancient time, drugs have been delivered though the nasal cavity for local and systemic drug delivery. For instance, in *Ayurveda*, the traditional Indian medicine, *Nasya* therapy, one of the *Panchakarmas*, is the process by which a medicine (in form of decoctions, oils and fumes) is intranasally administered [11].

**Figure 2:**
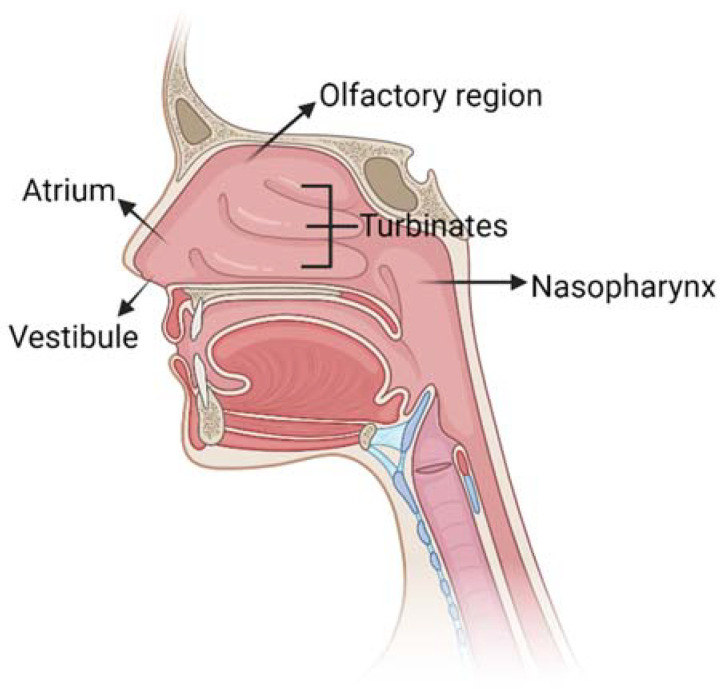
Schematic representation of nose anatomy with its five main regions: vestibule, atrium, turbinates in the respiratory region, olfactory region and nasopharynx. Created with BioRender.com.

The nose allows the entrance of air into the body during respiration: its main function is to filter, warm and humidify air. Moreover, the nose provides an immunological barrier to protect the nasal cavity from irritations and infections and it is the seat of the olfactory sense. The nasal cavity has a total volume of 16–19 mL [12] and it is composed of five main regions (Figure 2): the vestibule, the atrium, the olfactory region, the respiratory zone and the nasopharynx. 

**Figure 3:**
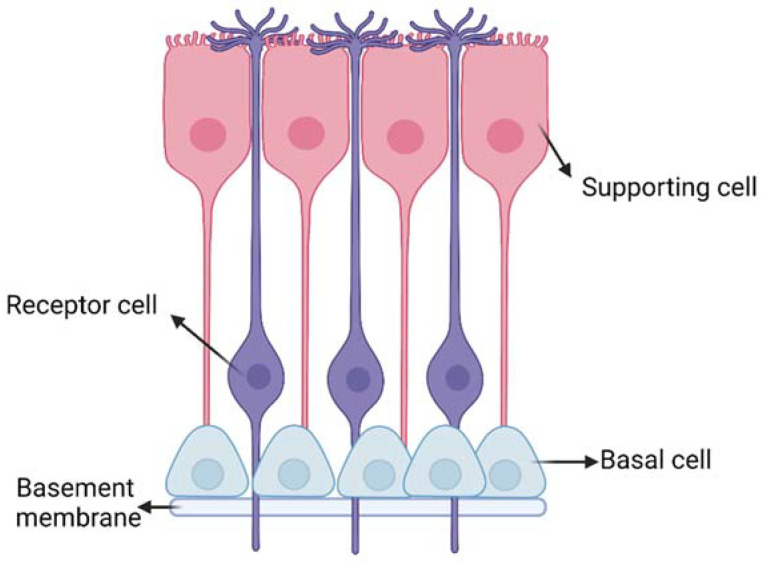
Schematic representation of the olfactory epithelium with the three main cells: (i) the olfactory receptor cells provided with non-motile cilia; (ii) the supporting cells and (iii) the basal cells. Created with BioRender.com.

The respiratory and olfactory regions are the ones involved in IN drug delivery due to their superior permeability and vascularization compared to the other nasal sites. The respiratory and olfactory regions have an extent of around 160 cm^2^ and 15 cm^2^, respectively [5]. The olfactory epithelium is composed of different cells (Figure 3): (i) the olfactory receptor cells provided with non-motile cilia; (ii) the supporting cells and (iii) the basal cells [13]. Moreover, it is coated with a mucus layer produced by the Bowman’s glands. The olfactory receptor cell is a bipolar neuron, forming an amyelinated axon at its basal surface, conveying olfactory information to brain. In its apical surface, each olfactory receptor develops a unique dendritic process that expands into a protuberance with several microvilli called olfactory cilia. Olfactory cilia are coated with a mucus layer: when, during a cold, the mucus layer thickens, olfactory sensibility decreases [14]. Amyelinic fibers and blood vessels run along the basal part of the mucosa, called lamina propria.

**Figure 4:**
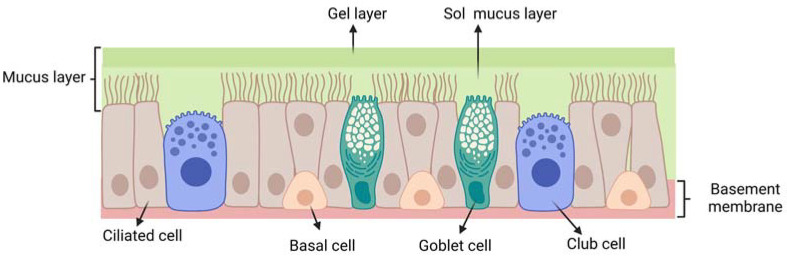
Schematic representation of the respiratory epithelium: ciliated pseudostratified columnar epithelial cells; club cells (non-ciliated cells); goblet cells; mucus layer (gel layer); sol mucus layer; basal cells; basement membrane. Created with BioRender.com.

The respiratory epithelium (Figure 4) is composed of: (i) ciliated pseudostratified columnar epithelial cells (with around 100 cilia per cell); (ii) non-ciliated cells; (iii) goblet cells and (iv) basal cells. Each ciliated and non-ciliated cell possesses around 300 microvilli [15]. The respiratory epithelium is coated with a mucus layer and its cilia are responsible for muco-ciliary clearance, the protective mechanism of the respiratory system. 

## 3. Mechanism for Drug Delivery to the Brain through the Intranasal Route

### 3.1. Mucus Layer: A First Barrier to Intranasal Drug Delivery

A first barrier for intranasal drug transport is represented by the mucus layer on the nasal epithelium. Mucus, mainly composed by mucin polysaccharide, is a hydrogel layer with intrinsic porosity and negative charges. Such features may slow down the drug diffusion rate through the mucus layer. When the drug diffusion rate across the mucus layer is lower compared to the muco-ciliary clearance rate, drug bioavailability decreases. It has been estimated that the muco-ciliary clearance half-time is around 15–30 min [16,17]. Clearance may be counteracted by the use of muco-adhesive drug carriers, as described in Section 5.

Furthermore, the nasal cavity has a slightly acidic pH (5.5–6-5) and contains enzymes that may catalyze the degradation of drugs, such as peptides and proteins, thus reducing drug bioavailability [18]. Typical approaches to address such issues are the use of enzyme inhibitors or drug doses above the saturation concentration of enzymes [19].

### 3.2. Transport of Drugs from Nasal Epithelium to the Brain

The precise pathways and mechanisms by which a drug travels from the nasal epithelium to various regions of the brain have not been fully elucidated. Lochhead et al. [5] and Thorne et al. [20] have described such mechanisms. Studies on [^125^I]-labeled proteins following IN administration in rats and monkeys have shown that delivery occurs along the olfactory and trigeminal nerve components in the nasal epithelium to the olfactory bulb and brainstem, respectively, with further dispersion to other brain areas [20]. At least three sequential transport steps are necessary for drugs to be delivered to the brain following IN administration [5]. 

#### 3.2.1. Transport across the Olfactory and Respiratory Epithelial Barriers

Transport across the olfactory or respiratory epithelia may occur either by intracellular or extracellular mechanisms. 

Intracellular pathways are activated by the olfactory sensory neurons, the trigeminal nerve and the nasal epithelial cells and include two different mechanisms depending on the involved cells: (i) endocytosis by the neural cells on the nasal epithelial surface and subsequent intraneuronal transport; (ii) transcytosis (i.e., transcellular transport) across the cells of the respiratory and olfactory epithelium to the lamina propria.

Extracellular transport pathways include paracellular diffusion across the olfactory and the respiratory epithelia to the underlying lamina propria. Paracellular transport depends on the presence of tight junctions in the olfactory and respiratory epithelia: tight junction tightness and continuity determine the permeability to paracellular transport. It has been suggested that the regular turnover of cells in the nasal epithelium may lead to continuous rearrangement and loosening of tight junctions which favors paracellular transport of drugs [5]. 

#### 3.2.2. Transport from the Nasal Mucosa to the Sites at Brain Entry

This transport may occur via intracellular pathways (intraneuronal transport through endocytosis within olfactory sensory neurons or trigeminal ganglion cells) or extracellular pathways (diffusion or convection within perineural, perivascular or lymphatic channels, associated with olfactory or trigeminal nerve bundles extending from the lamina propria to the brain). In the case of substances reaching the extracellular space, different fates are possible: (i) absorption into blood vessels, entering the systemic circulation; (ii) absorption into lymphatic vessels reaching neck cervical lymph nodes; (iii) extracellular diffusion or convection in perineural or perivascular nerve bundles spaces, leading to access to the cranial site. Drugs absorbed into the systemic circulation should cross the BBB or blood–cerebrospinal fluid (CSF) barrier to reach the brain. Hence, the nasal vasculature may act as a sink hindering some molecules from reaching the brain. Drugs that have reached the lamina propria and escaped the local absorption into the blood stream and drainage within nasal lymphatics may enter the brain tissue. Studies have shown that drugs can be transported in the spaces of the perineural sheath surrounding the olfactory nerve [5]. Moreover, although tight junctions are present, olfactory ensheathing cells maintain open spaces for the regrowth of olfactory nerve fibers, creating an additional extracellular path that substances may take to reach the brain, along with entering the olfactory nerve bundles. Finally, the perineural spaces of cranial nerves, such as the olfactory and trigeminal nerves, appear to allow communication with CSF of the subarachnoid space for some substances, providing an additional route for molecules to reach the brain [5].

#### 3.2.3. Transport from the Initial Brain Entry Sites to Other Brain Areas

Once the drug has reached the olfactory bulb and brainstem, it is distributed to other brain areas by two distinct possible mechanisms: (i) intracellular transport, by drug transfer to neurons forming a synapsis with peripheral olfactory sensory neurons or trigeminal ganglion cells; (ii) extracellular transport, through distribution within the cerebral perivascular spaces into the parenchyma. For instance, in the case of [^125^I]-labeled proteins such as insulin-like growth factor 1 (IGF-1) and interferon-β1b (INF-β1b), a convective mechanism within perivascular spaces of the cerebral blood vessels caused their distribution to different brain sites [20]. Expansion and contraction of perivascular spaces with the cardiac cycle may generate a pronounced fluid flow within them under normal conditions. Although modeling studies have been performed to understand the direction and characteristics of this flow, different results have been obtained and predictions of drug distribution in the brain are still a challenge [21,22]. However, increased blood pressure and heart rate have been demonstrated to improve the intraparenchymal distribution of large substances via the perivascular spaces [23]. Another study has also found that the rostral migratory stream, the pathway used by neuronal progenitors to migrate from perivascular regions to the olfactory bulb, may also play a role in the delivery of molecules from the nasal cavity into the brain [24].

Among the possible transport mechanisms to the brain, the direct axonal transport has been found to be incompatible with the short measured time taken by intranasally administered drugs to reach the brain [25]. Such findings have demonstrated that direct axonal transport is not the main route for IN drug delivery to the brain.

### 3.3. Synthesis of the Main Features of Intranasal Drug Transport to the Brain

**Figure 5:**
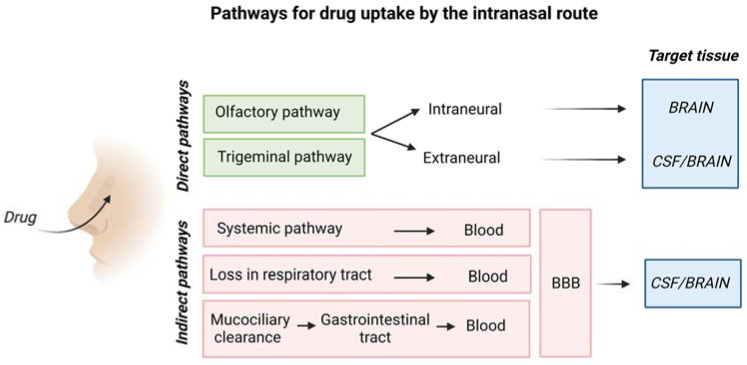
Schematic representation of drug uptake by the intranasal route. Created with BioRender.com.

Figure 5 is a schematic representation of the overall mechanism for IN drug delivery to the brain.

The olfactory pathway conveys drugs directly to the olfactory bulb, whereas the trigeminal nerve enters the CNS in the pons transporting drugs to cerebrum and cerebellum [26].

Table 1 describes advantages and disadvantages of the IN drug delivery route.

The degree of drug targeting to the brain after intranasal (IN) administration with respect to intravenous (IV) administration can be assessed by the drug targeting efficiency (DTE %) and the direct transport percentage (DTP %) [26]:

(1) $$\mathrm{DTE}\%=\left(\frac{\left(\frac{{\mathrm{AUC}}_{\mathrm{brain}}}{{\mathrm{AUC}}_{\mathrm{blood}}}\right)\mathrm{IN}}{\left(\frac{{\mathrm{AUC}}_{\mathrm{brian}}}{{\mathrm{AUC}}_{\mathrm{blood}}}\right)\mathrm{IV}}\right)\times 100$$

(2) $$\mathrm{DTP}\%=\left(\frac{B_{\mathrm{IN}}-{\;B}_{X}}{B_{\mathrm{IN}}}\right)\times 100$$

AUC is the area under the drug concentration–time curve.

B_IN_ is the AUC_0–24h_ (brain) following intranasal administration.

B_X_ is the brain AUC fraction from systemic circulation after intranasal administration.

(3) $$B_{X}=\left(\frac{B_{\mathrm{IV}}}{P_{\mathrm{IV}}}\right)\times P_{\mathrm{IN}}$$

B_IV_ is the AUC_0–24h_ (brain) following intravenous administration.

P_IN_ is the AUC_0–24h_ (blood) following intranasal administration.

P_IV_ is the AUC_0–24h_ (blood) following intravenous administration.

DTE% is used to indicate the tendency of the drug to accumulate in the brain when administered via the IN route compared to IV administration. Values above 100% evidence a higher efficacy of the IN compared to IV route. DTP% refers to the quantity of drug entering the brain through direct pathways (i.e., trigeminal and olfactory pathways). When DTP% is higher than zero, drug brain targeting is reached via the direct pathways, while for values below 0 the IV route is more effective compared to IN administration. Finally, a DTP% of 100 can only be achieved if the drug is not adsorbed by the blood circulation after IN administration (i.e., P_IV:_ AUC_0–24h_ (blood) is zero) or if the drug is not able to cross the BBB (i.e., B_IV_: AUC_0–24h_ (brain) is zero) [27,28].

Alternatively, the percentage of drug accumulation in the brain (bioavailability, B%) can be evaluated considering only the AUC data for the brain areas, without including AUC for blood:

(4) $$B\%=\left(\frac{{\mathrm{AUC}}_{\mathrm{brain}\;\mathrm{IN}}}{{\mathrm{AUC}}_{\mathrm{brain}\;\mathrm{IV}}}\right)\times 100$$

As for the DTE, a higher accumulation of the drug in the brain is represented by B% values above 100.

When nanocarriers are employed, the relative bioavailability (RB%) can be evaluated, comparing the IN drug delivery through nanoparticles with respect to the delivery of the drug in solution form.

(5) $$\mathrm{RB}\%=\left(\frac{{\left({\mathrm{AUC}}_{\mathrm{brain}\;\mathrm{IN}}\right)}_{\mathrm{nanosystem}}\;}{{\left({\mathrm{AUC}}_{\mathrm{brain}\;\mathrm{IN}}\right)}_{\mathrm{solution}}\;}\right)\times 100$$

Values of RB% above 100 indicate a higher accumulation of drug in the brain with the use of the nanocarriers compared to the “naked” drug. 

Moreover, RDTE% and RDTP% can also be utilized to evaluate the effect of the nanocarriers in drug administration through the IN route.

(6) $$\mathrm{RDTE}\%=\left(\frac{{\left({\mathrm{DTE}}_{\mathrm{IN}}\right)}_{\mathrm{nanosystem}}}{{\left({\mathrm{DTE}}_{\mathrm{IN}}\right)}_{\mathrm{solution}}\;}\right)\times 100$$

(7) $$\mathrm{RDTP}\%=\left(\frac{{\left({\mathrm{DTP}}_{\mathrm{IN}}\right)}_{\mathrm{nanosystem}}}{{\left({\mathrm{DTP}}_{\mathrm{IN}}\right)}_{\mathrm{solution}}}\right)\times 100$$

It has been found that an efficient delivery to the brain by the intranasal route also depends on the head position, type of used formulation and delivery device [10].

### 3.4. Prevalent Transport Mechanisms for Hydrophilic Drugs by the Intranasal Route

Intranasally administered drugs mainly cross the nasal epithelium barrier by a transcellular or paracellular mechanism (rather than by direct axonal transport) and, then, a portion of them is successfully transported to the brain, by diffusion in the perivascular or perineural spaces along the olfactory or trigeminal nerves. However, transport mechanisms depend on drug chemistry. A paracellular mechanism is possible for polar drugs with molecular weight lower than 1000 Da, allowing the diffusion thorough the 10 Å sized channels of transiently opened tight junctions in the nasal epithelium [29]. A polar drug with molecular weight higher than 1000 Da, such as polypeptides and proteins, can pass the nasal membrane by endocytic transport, although in low amounts [29]. For high molecular weight hydrophilic drugs, absorption enhancers can be used, including surfactants, bile salts and their derivatives, fatty acids and their derivatives, phospholipids, various cyclodextrins and cationic molecules (e.g., poly(lysine) or chitosan and its derivatives) [29], as explained in detail in the next section. Briefly, absorption enhancers increase the permeability of the epithelial cell membrane by different mechanisms depending on the type of enhancer, for example, increasing transcellular transport [29]. Moreover, they can also improve paracellular transport by promoting the transient opening of tight junctions. However, due to their interaction with the nasal epithelium layer, absorption enhancers can elicit cytotoxic effects towards the nasal mucosa, especially in the case of repeated drug administration for the treatment of chronic diseases [30,31].

## 4. Penetration Enhancers in IN Drug Delivery

Penetration enhancers have been proposed for IN drug delivery formulations: they can be components of the drug carriers or additives of the drug formulations, and can be used alone or in combination to exploit synergistic effects. A range of surfactants have been investigated using both synthetic (e.g., sodium lauryl-sulfate) and naturally derived (e.g., BC9-BS biosurfactant isolated from *Lactobacillus gasseri BC9*) materials to improve drug penetration by inducing membrane rupture or improving the fluidity of the membrane, then favoring transcytosis [32,33]. Non-ionic alkyl saccharide surfactants, including dodecyl maltoside (Intravail^®^), have also been associated with the possibility to enhance drug penetration by transcellular transport involving cellular internalization of drugs into vesicles, although the mechanism of action is still under investigation [34]. Nonetheless, dodecyl maltoside is commercially available in US FDA-approved nasal sprays for migraine (Tosymra^®^) and seizure clusters (Valtoco^®^).

Cationic polymers (e.g., chitosan or poly-L-arginine) have been shown to interact with the negatively charged cellular residues, reducing transepithelial resistance and promoting tight junction opening, which affects paracellular transport [35,36]. Tight junctions opening can also be favored by a reduction of endogenous calcium ions through the use of calcium chelators (e.g., ethylenediaminetetraacetic acid, EDTA) or anionic poly(acrylic acid) polymers (e.g., Carbapol^®^) able to bind cations [33,37]. Finally, the use of peptide-mimicking toxins has also been investigated to modulate the function of tight junctions. The C-terminal fragment of an enterotoxin derived from the Gram-positive bacterium *Clostridium perfringens* (C-CPE) has been shown to increase paracellular transport of nasal pneumococcal vaccine by binding with claudins, proteins involved in tight junction formation [38,39]. The peptide AT1002 is an analog of Vibrio cholera toxin, acting on *zonula occludens* in tight junctions. Peptide AT1002 enhances IN permeation by binding its receptor reversibly and opening tight junctions [40]. 

Mucolytic agents constitute another class of penetration enhancers for IN delivery able to decrease the viscosity of mucus. N-acetyl-l-cysteine is a widely used mucolytic agent able to cleave the disulfide bonds responsible for mucin fiber crosslinking, increasing mucus mesh size and permeability [18]. 

## 5. Biomaterials-Based Vehicles for IN Drug Delivery 

Biomaterials-based delivery systems, consisting of microparticles, nanoparticles and hydrogels, offer the possibility to improve the efficiency of intranasal drug delivery to the brain, as they may protect drugs from degradation, increase their absorption into and transport across the nasal epithelium (while decreasing drug loss in the respiratory and digestive tracts during administration) and—in the case of tailor-designed nanoparticles—favor brain targeting. 

As mucus represents the first physical barrier for drug absorption by the nasal mucosa, specific chemical and physical features of the mucus layer have to be taken into account for the design of efficient IN drug delivery systems. A thin mucus layer of approximately 10–15 µm in thickness covers the nasal epithelium, consisting of a highly hydrated polymeric network secreted by the goblet cells in the mucosa, mainly composed by water (around 95 wt.%) and mucins (2–5 wt.%), glycoproteins containing sialic acid units. Previous studies have reported different values of mucus hydrogel mesh size (50–1800 nm), however, the average size of mucus intrinsic pores is considered to be 20–200 nm [41]. Besides water and mucins, mucus also contains various amounts of DNA, plasma proteins, immunoglobulins (particularly secretory IgA), lysozyme, lactoferrin, lipids and polysaccharides [41]. The pH of mucus is slightly acidic (pH 5.5–6.5) [42]. Furthermore, mucus is continuously propelled towards the pharynx by the cilia present in the respiratory mucosa, at a rate of 5 mm/min [43] with an average clearance time of around 15–30 min [16,17]. Muco-adhesivity, i.e., the ability to attach to the mucus, has been frequently proposed as a key feature for efficient polymeric drug carriers in the IN route. Different theories have been exploited to explain muco-adhesivity, as reviewed by Khutoryanskiy et al. [42]. In brief, muco-adhesion can arise from electrostatic interactions between positively charged materials in contact with negatively charged mucins (electronic theory). Additionally, hydrogen and van der Waals bonding or hydrophobic interactions can be established contributing to muco-adhesion (absorption theory). Based on the wetting theory, biomaterial adhesion to mucus layer is promoted by the ability of the drug formulation to wet and spread over the mucus layer. According to the diffusion theory, an interpenetration between macromolecules and the mucin network is responsible for muco-adhesion. Furthermore, the mechanical theory predicts that muco-adhesion depends on the contact area between the biomaterial and the mucus layer, which in turns depends on the carrier surface roughness. Finally, the fracture theory predicts that muco-adhesion is due to chemical compatibility between mucus and the biomaterial, leading to an interface resistant to relative detachment. Based on that, muco-adhesivity is typically a property of polymers with positively charged chains, such as chitosan, able to interact with negatively charged mucins or able to form hydrogen bonds or other secondary interactions (e.g., hydrophobic interactions) with mucins. On the other hand, non-adhesive materials include antifouling polymers, such as poly(ethylene glycol) (PEG). A review article by Sosnik et al. has discussed the main muco-adhesive polymers for drug delivery, including chitosan, cellulose derivates and poly(acrylic acid) and poly(methacrylic acid) derivatives [41].

### 5.1. Microparticles and Nanoparticles for IN Drug Delivery

**Figure 6:**
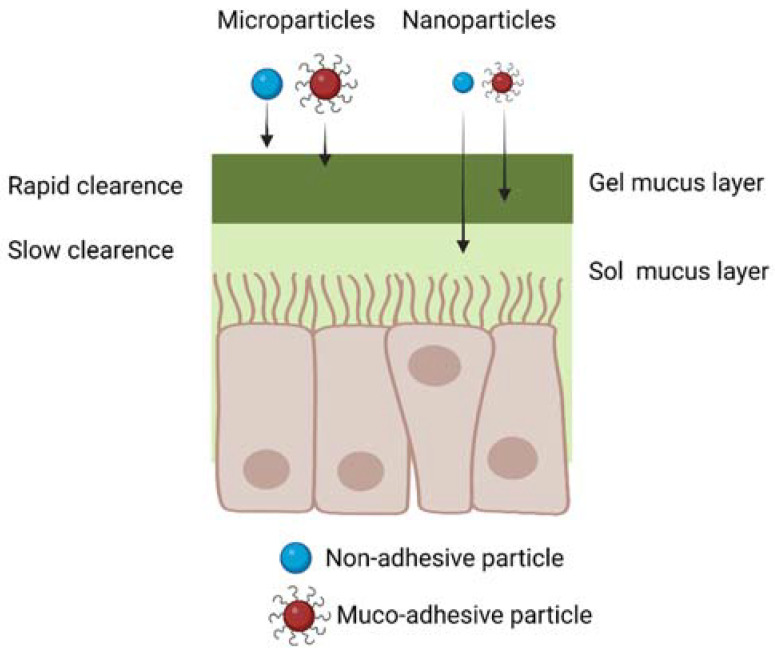
Schematic representation of micro- and nanoparticle diffusion through the nasal mucus considering both muco-adhesive and non-muco-adhesive particles. Created with BioRender.com.

Drug-loaded polymer microparticles, having sizes larger than 500 nm, are a suboptimal choice for IN drug delivery, as they are unable to diffuse through the smaller nanometric pores of the mucus layer (Figure 6). However, muco-adhesive microparticle with sizes in the 500 nm–10 µm range, escaping the filtration by the nasal vestibule, have been proposed for IN drug delivery, exploiting their ability to bind to the mucus layer [44,45]. Their drug delivery efficiency depends on the ability of loaded drugs to diffuse through the microparticles and the nasal mucus layer, reaching the nasal epithelium in a shorter time than that required for muco-ciliary clearance. Furthermore, in this application, drugs should be resistant to degradation and able to cross the nasal epithelium, without the help of biomaterial carriers.

On the other hand, drug-loaded nanoparticles are more advantageous than microparticles, due to their ability to penetrate and diffuse through the nasal mucus (Figure 6), and to support all the subsequent phases of drug transport to the brain, previously described in Section 3. Muco-adhesive nanoparticles have been frequently proposed for IN drug delivery, as they attach to the nasal mucus after administration, decreasing the possibility for their loss in the respiratory and digestive tracts [45,46]. On the other hand, muco-adhesive nanoparticles show limited drug delivery efficiency: as they slowly diffuse across the mucus layer, they are partially lost during the muco-ciliary clearance mechanism. As a solution, antiadhesive nanoparticles weakly interact with the mucus layer: although this property increases the quantity of nanoparticles lost in the digestive and respiratory systems post-administration, it increases nanoparticle diffusion rate across mucus. For this reason, antiadhesive nanoparticles have also been proposed for IN drug delivery [47,48,49,50].

Table 2 collects exemplary types of nanocarriers exploited for IN drug delivery.

Table 2 evidences that nanocarriers for IN drug delivery have been prepared from both natural materials and synthetic polymers. Among natural polymers, gelatin nanoparticles have been widely employed due to their biocompatibility, biodegradability, low immunogenicity and possibility for surface functionalization [70]. Frequently, gelatin nanoparticles have been prepared in combination with Poloxamer 188 to reduce mucus viscosity and elasticity, and modulate tight junction opening [58,71]. Chitosan nanoparticles have also been widely exploited for IN drug delivery [36,72]. Chitosan is a cationic polysaccharide able to induce tight junction opening, favoring paracellular transport of drugs when used in the form of nanoparticles, hydrogel or nasal solution [36,72].

Among synthetic polymers, poly(lactic-co-glycolic acid) (PLGA) copolymer has been widely investigated as a biocompatible and biodegradable material for the production of numerous drug delivery formulations, enabling encapsulation and controlled release of both hydrophobic and hydrophilic drugs [73]. Chitosan coating of PLGA-based nanoparticles has been mainly investigated to introduce muco-adhesive properties [55,56]. 

Lipid-based nanocarriers have also been investigated for IN drug delivery thanks to their low toxicity, biocompatibility, biodegradability and flexibility. In particular, second-generation lipid carriers, solid lipid nanocarriers (SLNs) and nanostructured lipid carriers (NLPs) have been mainly studied [56,62,63,69]. SLNs are composed of a solid lipid core containing the drug stabilized by surfactants, while NLPs possess a solid lipid core with a surfactant outer shell. SLNs and NLPs have shown higher drug loading, better drug stability and prolonged release profile compared to liposomes [74,75]. Finally, exosomes, naturally derived nanocarriers secreted by cells, have recently attracted interest as drug delivery systems for the treatment of brain disorders through IN administration thanks to their low immunogenicity, good biocompatibility, possibility of functionalization for targeted action and broad spectrum of endogenous and exogenous bioactive cargo molecules, including protein, nucleic acids, growth factors and therapeutic agents [65,67,76]. Interestingly, the nanocarrier surface has been frequently functionalized with:

- Muco-adhesive polymers such as chitosan or polymer containing thiol groups (thiomers);
- Molecules for adsorption endocytosis by the epithelial layer (e.g., lectins, cell-penetrating peptides, such as penetratin, Tat peptide, etc.);
- Molecules for ligand-mediated endocytosis (e.g., lactoferrin);
- Mucus-penetrating (non-adhesive) polymers (e.g., PEG).

Muco-adhesive functionalities have been used to decrease the muco-ciliary clearance time, increasing IN drug release efficiency. In some cases, the muco-adhesive molecules have additional functionalities, e.g., chitosan is able to enhance drug passage through the nasal mucosa by transiently opening the tight junctions connecting the epithelial cells as previously underlined. Thiomers have also been investigated for their muco-adhesive properties thanks to their interactions with cysteine residues of mucus glycoproteins [60,77].

Lectins are proteins able to reversibly bind mono-sugars or oligosaccharides, promoting both muco-adhesion and endocytosis [78,79]. Different types of lectins have been tested for surface functionalization of nanoparticles for IN drug delivery. Gao et al. reported the conjugation of WGA onto PEG-PLA particles [52,80], showing their enhanced brain targeting ability with respect to unfunctionalized particles, or intranasally or intravenously administered drug solutions. Gao et al. have also conjugated wheat germ agglutinin to PEG-PLA particles as it binds to L-fucose in the olfactory epithelium, enhancing drug release to the brain [52]. Chen et al. coupled STL to PLGA nanoparticles with a 1.89–2.45-fold increase in brain targeting [81]. STL-conjugated PEG-PLGA nanoparticles loaded with basic fibroblast growth factor (bFGF) enhanced brain delivery compared to intravenous injection of bFGF, and IN administration of bFGF solution and bFGF-loaded PEG-PLGA nanoparticles. The superior DTP % values of STL-conjugated PEG-PLGA nanoparticles indicated that more than 70% of the drug was directly delivered to the brain by the IN route [26].

Cell penetrating peptides have shown enhanced nose-to-brain transport [82,83]. Wan et al. have identified specific peptides for nose-to-brain drug delivery by the phage display method [82]. Yang et al. prepared liposomes loaded with rivastigmine and surface functionalized with a cell-penetrating peptide [64]. Zhai et al. modified the surface of exosomes with rabies virus glycoprotein (RVG) peptide able to specifically bind to neuronal acetylcholine receptor for the delivery of brain-derived neurotrophic factor for multiple sclerosis applications [67]. Finally, Peng et al. proposed a novel nanocarrier system, characterized by a polymer micellar core composed of PPS−PEG, encapsulated in an exosome outer shell modified with penetratin and RVG peptides for the delivery of curcumin for Parkinson’s disease treatment [66].

Lactoferrin is a natural protein binding iron, that has the ability to interact with the lactoferrin receptors that are abundant on respiratory epithelial cells. The functionalization of drug-loaded nanoparticles with lactoferrin allows their absorption by epithelial cells through a transcytosis mechanism. Liu et al. prepared PEG-co-poly(ε-caprolactone) nanoparticles surface functionalized with lactoferrin and incorporating NAP (NAPVSIPQ) a neuroprotective peptide [51].

As mentioned above, non-muco-adhesive properties have also been investigated to develop systems able to penetrate through the mucus barrier without adhering to it [49,50]. PEGylation is the main modification investigated to develop mucus-penetrating systems. However, the molecular weight and density of charge are two main parameters that affect the adhesion ability of PEG, as recently reviewed by Lai et al. [84]. Low molecular weight PEG combined with a high PEG density favors penetration, due to the generation of an almost neutral surface able to minimize mucus interactions. On the contrary, high molecular weight PEG and a low density of coatings have been shown to induce muco-adhesive properties [46,84]. Porfiryeva et al. showed the higher penetration of PEGylated derivatives of polyelectrolyte complexes formed by oppositely charged Eudragits (i.e., anionic Eudragit^®^ L100-55 and cationic Eudragit^®^ EPO) compared to non-modified complexes [54]. Ways et al. investigated the possibility to create mucus-inert chitosan nanoparticles through grafting with PEG, PHEA, POZ and PVP. All the modified nanocarriers showed superior mucus penetration compared to unmodified chitosan with PVP showing the highest penetration depth in ex vivo sheep nasal mucosa [48]. 

### 5.2. Hydrogels for IN Drug Delivery

Hydrogels are three-dimensional crosslinked networks of hydrophilic polymers able to absorb and retain a considerable amount of water without dissolving, preserving their shape [85]. Hydrogels for IN drug delivery have been generally designed to be muco-adhesive in order to bind to the nasal mucosa. Hydrogels may also undergo some mixing with the mucus layer, facilitating drug penetration [86]. Moreover, IN administration of hydrogels affects the viscosity of the mucus–hydrogel systems, increasing the muco-ciliary clearance time and enhancing the effectiveness of IN drug uptake [87]. Finally, hydrogels can shield drugs from undergoing chemical and enzymatic degradation in the nasal cavity, prolonging their activity. 

Hydrogels are generally sprayed into the nasal cavity or administered as solution drops, converting into a hydrogel upon contact with the nasal mucosa. Stimuli-responsive hydrogels have been generally used for IN drug delivery exploiting the following physical features of the nasal cavity: (1) pH 5.5–6.5; (2) temperature of 32 °C; (3) presence of sodium, calcium and potassium ions [86]. Hence, thermosensitive and pH- and ion-responsive hydrogels have been proposed for IN drug delivery as reviewed by Chonkar et al. and Protopapa et al. [86,88].

Table 3 collects exemplary types of promising hydrogel formulations for drug delivery to the brain by the IN route developed to date.

### 5.3. Nanocarrier-Loaded Hydrogels for IN Delivery

As discussed in Section 5.1, both antiadhesive and muco-adhesive nanoparticles have been proposed for IN drug delivery. However, the potential advantages of muco-adhesive nanoparticles have not been confirmed by in vivo biodistribution trials. Indeed, studies in mouse models have shown that muco-adhesive chitosan-coated nanostructured lipid nanocarriers, loaded with protein drugs, although effective for drug delivery to the brain, mainly accumulated in the lungs, followed by the liver, the kidneys, the spleen and, finally, the brain [68]. These findings have prompted criticisms on the real benefits of muco-adhesive nanoparticles for IN drug delivery to the brain, as such nanoparticles could more easily reach different tissues with potential side effects.

To overcome these drawbacks, more complex pharmaceutical formulations could be exploited, based on hydrogels releasing drug-loaded nanoparticles. Both weakly muco-adhesive hydrogels, such as Poloxamer [98,99], and highly muco-adhesive hydrogels, such as chitosan, Poloxamer/gellan and Poloxamer/chitosan [100,101,102,103,104], have been proposed. Such formulations are collected and described in Table 4. The hydrogel should improve local nanoparticle retention, decreasing losses in the digestive and respiratory tracts. Furthermore, hydrogels may increase the muco-ciliary clearance time and improve nanoparticle uptake by the nasal epithelium. 

Although different types of nanoparticles have been administered in combination with hydrogels, nanoparticles based on lipids and/or natural polymers are affected by limited stability and can be more easily disassembled/degraded in vivo with respect to nanoparticles based on synthetic polymers [105,106]. Hence, the type of hydrogel embedding nanoparticles should be carefully selected to avoid unfavorable interactions with lipid- or natural polymer-based nanoparticles, leading to their disaggregation in contact with the hydrogel.

**Figure 7:**
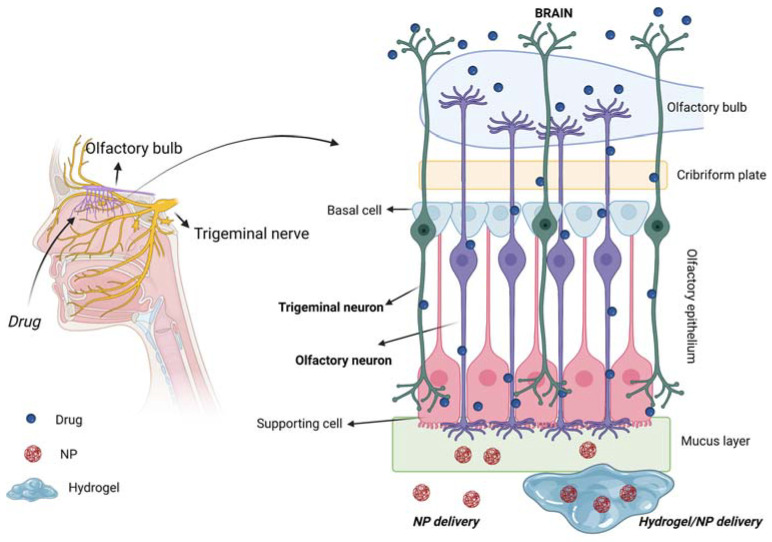
Scheme of IN drug delivery to the brain following the two direct olfactory and trigeminal pathways for drug uptake by the brain. Created with BioRender.com.

Figure 7 schematically shows the routes for drug transport to the brain of intranasally administered drugs, mediated by the use of the above-described biomaterials (i.e., various types of NPs, hydrogels and NP/hydrogel systems). The olfactory and trigeminal pathways are highlighted as being the two main direct routes for drug delivery to the brain. 

## 6. Discussion

IN delivery represents a direct route for effective drug administration to the brain, particularly suitable for hydrophilic high molecular weight drugs, such as growth factors, which are not able to cross the BBB and can induce side effects in the peripheral tissues when systemically administered. 

Kozlovskaya et al. analysed 73 publications between 1970 and 2014 concerning the quantitative analysis of the delivery of drugs or model agents to the brain via IN and parenteral routes. According to this literature investigation, the use of nanoparticles or hydrogel carriers for IN drug delivery has offered limited advantages with respect to drug solutions. On the other hand, compounds able to favor drug delivery through the IN route, such as absorption enhancers muco-adhesive compounds and targeting ligands have shown a higher impact on drug delivery effectiveness of IN therapy [107]. 

Particularly, drug-loaded nanoparticles have a key role in protecting drugs from enzymatic degradation and in efficiently transporting them across the nasal barrier to the brain through proper surface functionalities. Drugs crossing the nasal barrier are partially absorbed by the systemic circulation and hence not able to reach the brain (Figure 5). Their encapsulation into intranasally administered nanoparticles, provided with the additional ability to cross the BBB, could increase the efficiency of drug release to the brain. Additionally, nanoparticles could be functionalized with specific ligands potentially favoring drug release to specific brain cells. Among nanoparticles prepared from natural polymers or their derivatives (Table 2), chitosan-based nanoparticles have been widely used for IN delivery due to their muco-adhesive properties [36,72]. However, they do not have the intrinsic ability to cross the BBB or to reach target brain cells, unless properly functionalized [108]. Due to their hydrophilicity and consequent limited stability in physiological media, chitosan nanoparticles cannot be easily surface functionalized after their preparation. On the other hand, surface functionalization could be achieved through complex and laborious methods involving chemical derivatization of chitosan molecules before nanoparticle preparation [109]. One additional drawback of chitosan nanoparticles for protein or polypeptide drug encapsulation is the use of acidic solution for their preparation, with the risk of drug denaturation/degradation [110]. Similarly, nanocarriers provided with a lipid shell and a gelatin core are among the most widely exploited for IN drug delivery: they can be prepared in mild conditions and the lipid shell favors cell internalization (Table 3) [57,58]. However, their stability could be limited by rapid in vivo disassembly and gelatin degradation by proteolytic enzymes [75,111,112]. Unfunctionalized lipid–gelatin nanoparticles have been generally used for IN drug delivery, however, their surface functionalization with targeting ligands could be achieved by the use of previously synthesized ligand-functionalized lipids [113]. PEG-polyester nanocarriers (Table 2) represent another class of nanoparticles widely used for IN drug delivery, due to their biocompatibility, biodegradability and antifouling properties [51,52]. However, they also show a few limitations when applied for the release of protein drugs: (i) proteins can be loaded into PEG-polyester nanoparticles by emulsion methods, using organic solvents, which may affect protein bioactivity; (ii) surface functionalization of PEG-polyester nanoparticles with targeting ligands generally requires their incubation into functionalizing solutions, potentially causing partial protein drug release and/or denaturation/degradation; (iii) degradation of PEG-polyester nanoparticles by bulk hydrolysis may cause denaturation/degradation of loaded proteins before they reach their therapeutic target [114]. 

Overall, state-of-the-art analysis carried out in this review paper has shown that optimal nanocarriers for IN delivery of drugs (particularly proteins and polypeptides) to the brain have not yet been designed. Requirements for optimal nanoparticles for IN drug delivery to the brain include: high encapsulation efficiency, preparation using mild conditions, suitable size for diffusion through the intrinsic pores of the mucus layer, stability in contact with mucus and blood, antifouling surface functionalities (to facilitate diffusion through the mucus) coupled with specific ligands favoring transport through the nasal epithelium and subsequent brain targeting. One additional requirement is nanoparticle stability in the presence of an additional hydrogel exploited to increase IN drug delivery efficiency. Indeed, the combination of drug-loaded nanocarriers and hydrogels could enhance drug absorption by the nasal mucosa, decreasing the amount of nanocarriers lost in the gastrointestinal and respiratory systems. However, previous research studies have suggested that hydrogels need to be properly designed to avoid the risk of slowing down the diffusion ability of polymer nanocarriers to reach the nasal epithelium, with a consequent decrease in nanocarrier uptake [115]. Intermolecular interactions between the nanocarrier surface and the hydrogel should be weak while hydrogel (as well as hydrogel/mucus) mesh size should be larger compared to nanocarrier diameter. 

Based on our recent research work (unpublished), we have found that polymer nanocarriers with a neutral surface are generally stable and undergo sustained release from hydrogels at a rate depending on nanocarrier–hydrogel reciprocal interactions and mesh size. Conversely, positively charged nanocarriers should be embedded in hydrogels with neutral charge to avoid their collapse within the hydrogel with consequent premature cargo release. Hydrogel composition should also be selected considering its possible positive effect as an enhancer for nanocarrier uptake by the nasal epithelium.

More broadly, the design of efficient systems for IN drug delivery to the brain should ensure:

1. Therapeutic concentrations of drugs to the brain within a short time from the administration (~30 min), with prompt therapeutic benefits for the patients.
2. Improved drug bioavailability avoiding hepatic first pass metabolism.
3. Reduced side effects due to the lack of accumulation into non-target tissues, such as the liver, or the possibility to avoid gastroprotective drugs (needed for orally administered drugs in the treatment of chronic diseases).
4. Patient-compliant treatment exploiting a minimally invasive route.
5. Reduction of therapy costs due to enhanced effectiveness including brain-targeting ability.
6. Avoidance of local and systemic toxicity, which is frequently associated with long-term treatment of chronic diseases.
7. Improvement of patient’s quality of life, by an effective drug administration route.

IN therapy could be applied for the treatment of several brain pathologies, including neurodegenerative diseases, stroke and brain tumors. To date, IN drug formulations for specific treatment of brain pathologies are missing on the market or still at the stage of clinical trials. One major example is represented by the IN drug delivery of insulin, investigated in the SNIFF clinical trials as a possible treatment for cognitive defects in patients affected by Alzheimer’s disease (clinicaltrials.gov: NCT03857321, NCT00438568, NCT01767909). Clinical trials have shown side effects of IN delivery of insulin formulations, such as nosebleeds and rhinitis [116]. Indeed, all the tested insulin formulations (Detemir, Humalog, Apidra, etc.) made use of excipients, such as cresol, meta-cresol and phenol, responsible for rhinitis, nosebleeds and allergic reactions [117]. Additionally, insulin formulations have shown effectiveness in improving the cognition of adults with mild cognitive impairment or early-stage Alzheimer’s disease [118]. On the contrary, they have failed to impact on cognition in individuals with mild–moderate Alzheimer’s disease carrying the ε4 allele of apolipoprotein E, an established risk factor for late-onset Alzheimer’s disease [119,120]. These findings suggest that although insulin may have some therapeutic effect in the Alzheimer’s disease treatment, improved IN insulin delivery formulations are needed. Phase 2 and 3 controlled trials have been recently completed, investigating the efficacy of two IN insulin devices (ViaNase by Kurve Technology and I109 Precision Olfactory Delivery by Impel NeuroPharma) on patients with mild cognitive impairment or Alzheimer’s disease over 18 months. Preliminary results suggested a better performance of the ViaNase device, showing higher cognitive performance scores and Alzheimer’s disease biomarker production. However, possible differences in the dosage delivered by the two systems might have led to the discrepancies in the results [121,122], leading to the set-up of a new clinical trial, currently ongoing, to evaluate the specific dosage of insulin delivered by the devices (SNIFF Device, clinicaltrials.gov: NCT01767909). 

One main problem in the design of intranasal drug delivery formulations is their preclinical validation through poorly predictive in vivo mouse and rat models [123,124]. Recently, physiologically relevant in vitro models of the human nasal epithelium have been developed and are commercially available for preclinical investigations. For example, EpiNasal™ by MatTek Life Sciences mimics the nasal epithelium structure by an air–liquid interface coculture of epithelial cells and mucus-producing goblet cells with functional tight junctions and beating cilia. Moreover, Gholizadeh et al. developed a first nasal epithelial mucosa (NEM)-on-a-chip model able to measure in real time the barrier integrity (obtained through transepithelial electrical resistance, TEER, measurements) and the drug transport (e.g., ibuprofen transport, chosen as a model agent) across a human nasal cell layer cultured at the ALI interface in both static and dynamic (via pulsatile systemic circulation in the basolateral compartment) conditions [125]. Capuana et al. developed a perfusion bioreactor system mimicking the nasal mucosa, characterized by a scaffold with an inner channel allowing cells to be in contact with both cell culture medium and air under dynamic conditions [126]. However, micro- or milli-fluidic devices recapitulating drug transport through the nose to reach the brain would be required to improve the design of optimal drug formulations for IN drug delivery to the brain, allowing better preclinical validation studies and reduction in the use of animal experimentation, according to the principles of replacement, reduction and refinement (the 3Rs).

## 7. Conclusions

This review paper highlighted the advantages of IN drug delivery systems, for their potential ability to provide a direct, rapid and effective route for drug delivery to the brain for the treatment of cancer, neurodegenerative diseases, stroke and other pathologies (e.g., migraine). The knowledge of the routes taken by intranasally administered drugs, together with the main biological barriers involved in the transport, is fundamental for the design of biomaterials-based carriers able to efficiently deliver drugs to the brain. Although different delivery vehicles have been proposed, more research is needed to improve drug delivery efficiency, decreasing side effects, both locally and systemically. Multifunctional formulations based on hydrogels encapsulating drug-loaded nanocarriers could represent the solution and their composition should be optimized based on the physicochemical properties of drugs, nanocarrier release kinetics and optimal adsorption by the nasal epithelium. These efforts require the parallel development of improved in vitro models for accurate preclinical evaluation before clinical trials.

The increasing incidence of brain pathologies as a consequence of progressive aging makes urgent the need for effective IN drug delivery nanoformulations. IN drug delivery systems targeting the brain tissue have the potentiality to address societal challenges, providing effective treatments for age-related diseases, including neurodegenerative diseases and brain cancers. 

## Figures and Tables

**Table 1:** Summary of main advantages and disadvantages of the IN route for drug delivery to the brain.

Nasal Administration	
Advantages	Disadvantages
Rapid drug absorption	Possible irritation to the nasal mucosa especially for repeated administrations
Enhanced pharmacokinetics profile	Nasal cavity has smaller absorption surface area compared to the gastrointestinal tract
Drug degradation is limited with respect to oral administration	Risk for local side effects, e.g., irreversible damage to cilia.
Avoidance of first pass metabolism	Surfactants or penetration enhancers may elicit cytotoxic effects on nasal epithelial cells.
Direct brain targeting avoiding BBB crossing and possible systemic side effects of drugs	Possible partial loss of drug dose in the respiratory and gastrointestinal tracts during administration
Non-invasive and painless approach	Limited capacity of the nasal cavity (23 cm^3^ in humans receiving approximately 400 µL formulation) [10]
Patient compliance	Rapid muco-ciliary clearance

**Table 2:** Main nanocarriers exploited in IN drug delivery to treat brain pathologies.

Material	Surface Moieties	Drug	Size (nm)	Main Tests	Brain Targeting Yield *	Ref.
	**Nanoparticles based on synthetic polymers**					
Methoxy poly(ethylene glycol)-*co*-poly(ε-caprolactone) copolymer (Me-PEG-PCL, 15 kDa) and maleimide PEG-PCL copolymer (Mal-PEG-PCL, 18 kDa)	Lactoferrin (thiolated) (Lf)	Coumarin-6 (C6) or NAP (NAPVSIPQ), an 8-amino acid neuropeptide fragment Derived from the activity- dependent neuroprotective protein (ADNP) family	C6 based: from 73.2 ± 4.2 nm to 89.0 ± 5.7 nm With Lf and NAP-based: from 76.2 ± 6.5 nm to 88.4 ± 7.8 nm	Alzheimer’s disease mouse model obtained by intracerebroventricular co-injection of pre-aggregated Aβ_1–40_ and a small amount of ibotenic acid	AUC_brain_/AUC_blood_ for: Lf NPs = 2.69–3.51; NPs = 1.28–1.92	[51]
Methoxy PEG-*b*-poly(D,L-lactic acid-*co*- glycolic acid) copolymer (Me-PEG-PLGA) and maleimide PEG-b-PLGA (Mal-PEG-PLGA)	*Soranum tuberosum* lectin (STL)	Basic fibroblast growth factor (bFGF)	Non-functionalized: 104.8 nm Surface functionalized: 118.7 nm	As above	-	[26]
PEG-PLA	Wheat germ agglutinin (WGA)	Vasoactive intestinal peptide (VIP)	Non-functionalized: 90–100 nm Surface functionalized: 100–120 nm	Nasal biodistribution in male Sprague Dawley rats and Kunming mice (loading fluorescent probe 6-coumarin into the nanoparticles)	RB% for: WGA-NPs = 566–774; NPs = 357–474	[52]
PLGA (LA:GA 50:50)	Poloxamer 407	Diazepam (lipophilic drug to treat epilepsy)	From 148 ± 0.5 to 337 ± 1.8 nm; optimal size: 183.2 nm	Sprague Dawley rats using radiolabelled drug to detect biodistribution	DTE% = 258; DTP% = 61.3	[53]
Inter-polyelectrolyte complexes of Eudragit^®^ EPO (EPO) and anionic Eudragit^®^ L100-55 (L100-55) and PEGylated L100-55	-	Haloperidol (model psychoactive drug causing catalepsy in laboratory animals)	For EPO/L100-55: from 120 to140 nm For EPO/PEGylated L100-55: from 110 to 570 nm	Ex vivo retention in sheep nasal mucosa. In vivo retention studies in male Wistar rats	-	[54]
PLGA nanoparticles coated with chitosan	Chitosan	Carmustine (antitumor drug)	From 208 to 421 nm depending on formulation parameters	Glioblastoma treatment: ex vivo retention studies using goat nasal mucosa	DTE% = 687 ± 32; DTP% = 94 ± 3	[55]
PLGA	Chitosan	Meloxicam (Alzheimer’s drug)	142 ± 12.8 nm	Alzheimer’s disease treatment: no animal studies	-	[56]
	**Nanoparticles based on natural polymer**					
Gelatin nanostructured lipid carriers: gelatin, Poloxamer 188-grafted heparin, trehalose, cholesterol, glyceraldehyde crosslinker	Trehalose, cholesterol	Neuropeptide substance P (SP)	166.00 ± 1.32 nm (blank) 172.00 ± 1.52 nm (with SP)	In vivo trials in rats with 6-hydroxydopamine-induced hemi-parkinsonism	-	[57]
Gelatin nanostructured lipid carriers (gelatin core) (GNLs)	Poloxamer shell	Basic fibroblast growth factor (bFGF)	143 ± 1.14 nm	Parkinson’s disease treatment: in vivo trials in hemiparkinsonian rats	-	[58]
Chitosan (CS) crosslinked with tripolyphosphate (TPP) anions	-	Sumatriptan succinate, an antimigraine drug	306.8 ± 3.9 nm	Migraine therapy: animal tests not reported	-	[59]
Chitosan, glycol CS (GCS) and corresponding thiomer-based materials. TPP or sulfobutyl-ether-β-cyclodextrin (SBE-β-CD) crosslinking agents	Chitosan and thiomers	Dopamine	372 ± 81 nm for selected formulation (containing GCS and SBE-β-CD)	Parkinson’s disease treatment: experiments in rats	-	[60]
Chitosan grafted with PEG, poly(2-hydroxyethyl acrylate) (PHEA), poly(2-ethyl-2-oxazoline) (POZ) and poly(N-vinyl pyrrolidone) (PVP)	PEG, PHEA, POZ, PVP		Unmodified chitosan: 152 ± 13 nm PEG-chitosan: 137 ± 23 nm PHEA-chitosan: 142 ± 11 nm POZ-chitosan: 145 ± 21 nm PVP-chitosan: 130 ± 19 nm	No targeted disease: ex vivo penetration in sheep nasal mucosa	-	[48]
Chitosan (CS) crosslinked with TPP ions	-	Lurasidone hydrochloride, an antipsychotic drug	154.8 ± 4.5 nm	Schizophrenia treatment: ex vivo study of permeation in goat nasal mucosa	-	[61]
	**Nanoparticles based on lipids**					
Solid lipid nanoparticles: glyceryl monostearate, Pluronic 127 and Tween 80	-	Naloxone	190.2 nm	Opioid management: in vivo toxicity, biodistribution and pharmacokinetics studies in Sprague Dawley rats and New Zealand rabbits	AUC_0-t_ = 17.75 ± 1.08	[62]
Solid lipid nanoparticles: glyceryl dibehenate (i.e., Compritol^®^ 888 ATO) and Tween 80 and Poloxamer 188		Buspirone	218.60 ± 9.18 nm	Anxiolytic treatment: in vivo pharmacokinetic, biodistribution and brain targeting studies in albino Wistar rats	DTE% = 883; DTP% = 87	[63]
Solid lipid nanoparticles: phosphatidylcholine and Poloxamer 188	Chitosan	Meloxicam (Alzheimer’s disease drug)	94.8 ± 7.4 nm	Alzheimer’s disease treatment: no animal studies	-	[56]
Liposomes	Cell-penetrating peptide (CPP)	Rivastigmine	Unmodified liposome: 166.3 ± 17.4 nm Liposome/CPP: 178.9 11 ± nm	Alzheimer’s disease treatment: in vivo pharmacokinetic and nasal toxicity studies in male Sprague Dawley rats	-	[64]
Exosomes	-	Curcumin or signal transducer and activator of transcription 3 Stat3 inhibitor anti-inflammatory agents	135.9–205.3 nm	Brain inflammatory diseases in C57BL/6j mouse models: a lipopolysaccharide (LPS)-induced brain inflammation model; autoimmune encephalomyelitis disease model and GL26 brain tumour model		[65]
Micelles: Micellar core made of poly(propylene sulfide)–polyethylene glycol (PPS−PEG) Outer nano-shell layer based on mesenchymal stem cell-derived exosomes	Penetratin and rabies virus glycoprotein (RVG29) peptides	Curcumin and microRNA 133b	From 135.9 to 194.9 nm	Parkinson’s disease mouse model obtained by injection of 1-methyl-4-phenyl-1,2,3,6- tetrahydropyridine (MPTP)	-	[66]
Exosome	Rabies virus glycoprotein (RVG) peptide binding to neuronal acetylcholine receptor (nAchR)	Brain-derived neurotrophic factor	Around 100 nm	Multiple sclerosis: demyelination mouse model of C57BL/6 mouse model (cuprizone feeding)	-	[67]
Nanostructured lipid carriers: glyceryl distearate (Precirol ATO 5) or Dynasan 114 and Miglyol chosen to form lipid matrix	Chitosan	Human insulin-like growth factor 1 (IGF-1)	Precirol-based: from 72.1 ± 8.55 to 294.50 ± 22.06 nm Dynasal-based: from 127.87 ± 35.03 nm to 267.40 ± 2.12 nm	Neuroprotective and neurorestorative therapy in neurodegenerative diseases. In vivo toxicity and accumulation studies in C57 mice	-	[68]
Nanostructured lipid carriers: glyceryl monostearate (GMS) and oleic acid mixture and Tween 80	Chitosan	Buspirone (anxiolytic agent)	190.98 ± 4.72 nm	Anxiolytic treatment: in vivo pharmacokinetic and neuropharmacokinetic studies in albino Wistar rats	DTE% = 1462; DTP% = 93; RB% = 217 ± 13; B% = 306 ± 19	[69]

* Results of brain targeted yield were reported for studies where in vivo experiments were conducted and the data are described using the formulae reported in Section 3.3. AUC = area under the curve, DTE% = drug targeting efficiency, DTP% = direct transport percentage, B%= bioavailability, RB% = relative bioavailability.

**Table 3:** Main hydrogels exploited in IN drug delivery to treat brain pathologies.

Material	Muco-Adhesive Components	Drug	Main Tests	Brain Targeting Yield *	Ref.
	**Synthetic hydrogels**				
Poloxamer 407, Poloxamer 188	Carbapol 934P (CP) or chitosan	Rasagiline, an anti-Parkinson’s drug	Parkinson’s disease treatment: in vivo biodistribution in male Wistar rats and pharmacokinetic studies in female New Zealand white rabbits	Fold increase in B% for: CP-gel= 4.35; Chitosan-gel = 6.05	[89]
Poly(nisopropylacrylamide) (PNIPAM) and gelatin methacryloyl	-	Hydroxylated biphenol derived from the “Houpo” herb (*Magnolia officinalis*) known as magnolol (MAG)	Parkinson’s disease treatment: in vivo pharmacokinetics studies in male Sprague Dawley rats	DTE% = 810; DTP% = 88	[90]
Pluronic 407 and Pluronic 188 micelles	-	Rotigotine (dopamine agonists for Parkinson’s disease treatment)	Parkinson’s disease treatment: in vivo pharmacokinetics studies in male Sprague Dawley rats	B% = 84.6; DTE% = 201–327; DTP% = 49–69	[91]
Poloxamer 407, deacetylated gellan gum and sulfobutyl-cyclodextrin	-	Cinnarizine (Ca^2+^ channel blocker)	In vivo pharmacokinetics studies and distribution in male Wistar rats subjected to microwave-induced brain injury	DTE% = 116	[92]
Pluronic 407, Pluronic 188 and PEG 8000	-	Genipin (antidepressant-like potential)	Evaluation of antidepressant effects in male Institute of Cancer Research mouse model of reserpine-induced depression and pharmacokinetics studies in male Sprague Dawley rats	RB% fold increase = 2.13	[93]
	**Natural hydrogels**				
Chitosan with β-glycerophosphate	-	D-penicillamine, a water-soluble metal chelator	Alzheimer’s disease: in vivo studies in APPswe/PS1d9 double-transgenic mice and C57BL/6 mice	-	[94]
Chitosan with β-glycerophosphate	-	Exenatide (therapy for the treatment of type 2 diabetes)	Treatment of type 2 diabetes: in vivo biodistribution and pharmacokinetics in male SD rats, in vivo pharmacodynamics studies in male SD obesity rat model	RB% = 11.9 ± 0.89	[95]
Gelatin and hydroxypropyl methylcellulose (HPMC)	-	Rivastigmine tartrate (semisynthetic drug aganist moderately severe Alzheimer’s and Parkinson’s diseases)	Alzheimer’s disease and Parkinson’s disease treatment: no animal study	-	[96]
Gellan gum	Functionalization of gellan gum with primary amino groups	-	No targeted disease: ex vivo studies on adhesive properties using porcine small intestine mucus	-	[97]

* Results of brain targeted yield are reported for studies where in vivo experiments were conducted and the data are described using the formulae reported in Section 3.3. B% = bioavailability, RB% = relative bioavailability, DTE% = drug targeting efficiency, DTP% = direct transport percentage.

**Table 4:** Exemplary nanoparticle-loaded hydrogel formulations investigated for IN drug delivery to reach the brain.

Hydrogel Composition	Incorporated Nanocarriers	Drug	Treated Pathology	Reference
Chitosan or Carbopol 974 NF^TM^ in Poloxamer	Poly(amidoamine) dendrimers	siRNA	Parkinson’s disease, Alzheimer’s disease and brain tumors	[100]
Poloxamer 407 and Poloxamer 188	Solid lipid nanoparticles	Quetiapine fumarate	Schizophrenia	[98]
Poloxamer 127 and Poloxamer 68	Chitosan microspheres	Lorazepam (benzodiazepine derivative for the treatment of status epilepticus)	Status epilepticus	[99]
Poloxamer 407 and HPMC	Nanostructured lipid carriers	Rivastigmine (acetylcholinesterase inhibitor)	Alzheimer’s disease	[101]
Poloxamer 407 and chitosan	Silica nanoparticles	Curcumin	Alzheimer’s disease	[102]
Poloxamer 127/ Poloxamer 68, chitosan and guar gum	Pullulan nanoparticles	Eletriptan hydrobromide (antimigrane drug)	Antimigraine effect	[103]
Chitosan	Lipid nanoparticles	Temozolomide (chemotherapeutic)	Melanoma and glioma	[104]

## Data Availability

No new data were created or analyzed in this study. Data sharing is not applicable to this article.

## Abbreviation

ADNP	activity-dependent neuroprotective protein
AUC	area under the drug
B%	Bioavailability
BBB	blood brain barrier
bFGF	basic Fibroblast growth factor
C-CPE	*Clostridium perfringens*
CNS	central nervous system
CPP	Cell penetrating peptide
CS	Chitosan
CSF	blood-cerebrospinal fluid
DTE %	drug targeting efficiency
DTP %	direct transport percentage
EDTA	Ethylenediaminetetraacetic acid
GCS	glycol chitosan
GMS	Glyceryl monostearate
GNLs	Gelatin nanostructured lipid carriers
IGF-1	insulin-like growth factor 1
IGF-I	Insulin-like Growth Factor-I
IN	Intranasal
INF-β1b	interferon-β1b
Lf	lactoferrin
LPS	lipopolysaccharide
MAG	*Magnolia officinalis*
Mal-PEG-PCL	maleimide poly(ethylene glycol)-*co*-poly(ε-caprolactone) copolymer
Me-PEG-PCL	Methoxy poly(ethylene glycol)-*co*-poly(ε-caprolactone) copolymer
NP	nanoparticle
PEG	poly(ethylene glycol)
PHEA	poly(2-hydroxyethyl acrylate)
PLGA	poly(D,L-lactic acid-*co*-glycolic acid) copolymer
POZ	poly(2-ethyl-2-oxazoline)
PPS−PEG	poly(propylene sulphide)-polyethylene glycol
PVP	poly(N-vinyl pyrrolidone)
RB%	relative bioavailability (RB%)
RDTE%	relative drug targeting efficiency
RDTP%	relative direct transport percentage
SBE-β-CD	sulfobutyl-ether-β-cyclodextrin
SP	Neuropeptide substance P
STL	Soranum tuberosum lectin
TEER	transepithelial electrical resistance
TPP	tripolyphosphate
VIP	Vasoactive intestinal peptide
WGA	Wheat germ agglutinin
